# Adaptations to the welsh national exercise referral scheme during the COVID-19 pandemic: a qualitative study exploring the experiences of service users and providers and supplementary out-of-pocket cost analysis

**DOI:** 10.1186/s12889-025-21502-3

**Published:** 2025-02-01

**Authors:** Katie Newby, Neil Howlett, Adam P. Wagner, Nigel Lloyd, Imogen Freethy, Charis Bontoft, Olujoke Fakoya, Shelley Jackson, Carla Jackson, Wendy Wills, Mary-Ann McKibben, Annie Petherick, Katherine E. Brown

**Affiliations:** 1 Department of Psychology, Sport and Geography, School of Life and Medical Sciences, College Lane Campus, University of Hertfordshire, Room 1H273, C.P. Snow Building, Hatfield, AL10 9AB UK; 2 Norwich Medical School, University of East Anglia, Norwich Research Park, NR4 7TJ Norwich, UK; 3 National Institute for Health Research (NIHR) Applied Research Collaboration (ARC) East of England (EoE), Cambridge, UK; 4 Freedom Leisure, Powys, UK; 5 Merthyr Tydfil Leisure Trust, Merthyr Tydfil, UK; 6 Centre for Research in Public Health and Community Care, School of Health and Social Work, College Lane Campus, University of Hertfordshire, Hatfield, AL10 9AB UK; 7 Health Improvement Division, Public Health Wales, Cardiff, UK

**Keywords:** Physical activity, Exercise referral, Uptake, Adherence, Cost

## Abstract

**Background:**

Despite the proliferation of exercise referral schemes in the UK, evidence on their efficacy is equivocal. The Welsh National Exercise Referral Scheme (NERS) is heavily used but inequalities in uptake have been reported. As a result of the COVID-19 pandemic, NERS was initially suspended and then transitioned from standard face-to-face delivery to alternative remote methods, including virtual delivery. The aim of this study was to explore the barriers and facilitators to uptake and engagement of NERS when delivered in face-to-face and virtual formats, and to examine the cost to service users of engaging with the scheme in these different ways.

**Methods:**

This was a qualitative study with supplementary cost analysis. Maximum variation sampling was used to recruit participants. Interviews with service users (*n* = 21) and one person who declined the service, and three focus groups with service providers (*n* = 19), were conducted. Framework analysis was used to analyse the qualitative data. Quantitative data obtained through the interviews on service users’ out-of-pocket costs of attending face-to-face or virtual classes were summarised using descriptive statistics.

**Results:**

Five themes were identified from the qualitative analysis which summarised barriers and facilitators to uptake and engagement as perceived and experienced by the different stakeholders. Themes included: opaqueness and uncertainty around referral; Exercise Referral Professionals allaying concerns and providing reassurance at scheme entry; the mixed appeal and accessibility of virtual delivery; factors that support ongoing engagement; and personal and financial circumstances restricting uptake and engagement.

**Conclusions:**

This study indicates that offering a virtual version of NERS could make the scheme more accessible to those who are typically underserved, provided strategies to address digital inclusion are addressed. Findings provide wider evidence to inform adaptations that could be made to ensure that other exercise referral schemes can optimise virtual delivery.

**Supplementary Information:**

The online version contains supplementary material available at 10.1186/s12889-025-21502-3.

Participating in regular physical activity can improve a plethora of physiological outcomes important for the prevention of ill health (e.g., blood pressure, body composition, lipidaemia) [1]. Consequently, adherence to muscle strengthening and/or aerobic physical activity can reduce the risk of living with long-term conditions such as cardiovascular disease and cancer and is negatively associated with all-cause mortality [2]. Furthermore, physical activity is beneficial for the treatment and management of long-term conditions such as hypertension [3] cancer [4], coronary heart disease [5], and type 2 diabetes [6]. It is also well established that physical activity has positive associations with a range of mental health outcomes such as wellbeing [7, 8] and depression [9]. With these wide-reaching health implications, physical inactivity accounts for up to 1 in 6 deaths and is estimated to cost the United Kingdom (UK) £7.4 billion annually [10].

While public health authorities have been actively promoting physical activity for decades, adult physical activity levels worldwide indicate that 31% of people do not meet the guidelines of 150 min of moderate intensity physical activity per week [11]. In the UK, physical activity levels are consistently low [12]. This is true of Wales, where recent figures show that only 53% of those aged 16 + years meet the minimum guideline level of physical activity [13]. Further, certain groups in Wales are at increased risk of physical inactivity, including women, older adults, those living in deprived areas, those living with overweight or obesity, and those experiencing poor physical or mental health [13]. With higher rates of physical activity being a well-established public health objective in the UK, exercise referral schemes have been incorporated into both prevention and treatment pathways. Exercise referral schemes typically involve an assessment by a healthcare professional, referral to a physical activity specialist to determine an appropriate physical activity plan, and the chance to participate in several weeks of supported physical activity sessions [14]. The purpose is to support people to initiate and maintain long-term physical activity behaviour change.

Despite the proliferation of exercise referral schemes in the UK, they have shown mixed evidence in their ability to improve physical activity and wider wellbeing outcomes [15]. One review suggested that 17 inactive adults would need to be referred for one to become moderately active long-term [16]. Across systematic reviews, uptake (ranging from 35 to 81%) and attendance (ranging from 12 to 49%) show considerable variation but typically indicate high dropout and low engagement levels [17]. One of the issues with such a wide range of schemes offered, and the heterogeneous populations they serve, is the challenge of standardising delivery.

The Welsh National Exercise Referral Scheme (NERS) is the first standardised national exercise referral scheme. NERS is funded by the Welsh Government and centrally managed by Public Health Wales. The scheme, introduced in July 2007, operates in all 22 Local Authority areas of Wales and targets those aged 16 years and over, who are sedentary and are at risk of or with established disease. NERS aims to support service users to increase their confidence in engaging in regular physical activity and, in doing so, improve their physical and mental wellbeing. Understanding the barriers to uptake and engagement from both service user and provider perspectives is crucial to ensuring equitable uptake and benefit.

Studies have identified groups of individuals with potential access issues to NERS at different stages along the service user journey. In terms of referral and uptake, men, non-car users, those living in the most deprived areas, and those being referred for their mental health show lower uptake [18, 19]. This contrasts with higher uptake in older adults and those with long-term conditions such as musculoskeletal problems [19]. Trends in uptake are mirrored in engagement levels, with adherence lower among those that are younger, least active at the start of the programme, and/or living with mental health conditions [18]. A number of qualitative studies have explored factors influencing uptake and adherence to exercise referral schemes when delivered face-to-face. Facilitators include professional and social support, personalised sessions, accountability and intrinsic motivation [20–22]. Barriers include negative perceptions about the atmosphere and equipment in gyms, work commitments, childcare responsibilities, and location and cost factors [20, 21, 23]. To date, no studies have explored the barriers and facilitators of uptake and adherence to exercise referral schemes when delivered virtually.

In March 2020, NERS adapted to virtual delivery to support service users during the COVID-19 pandemic. This provided an opportunity to examine the impact of mode of delivery on scheme uptake and engagement from the perspective of service users and service providers. The number of referrals to NERS grows annually, such that demand risks outstripping capacity. Offering all or part of the scheme in a virtual format could have the potential to increase capacity and, therefore, the number (and type) of people who can be supported. However, although digital interventions can work to improve physical activity levels and associated health for target groups such as older adults [24], they may only work for those living with higher socio-economic status [25]. This is important to explore given that offering a virtual only version of the scheme could widen existing inequalities, with those on lower incomes more likely to be digitally excluded. There are also data to suggest that there may be a lower level of basic digital skills in Wales than the rest of the UK [26]. This research explores considerations around virtual delivery, enabling scheme commissioners and providers to make evidence-based decisions about future delivery. It also provides wider learning for a range of public health services that may have recently adapted to remote or hybrid delivery approaches or are considering doing so.

The aim of this study was to explore the barriers and facilitators to uptake and engagement of NERS when delivered in face-to-face and virtual formats, and to examine the cost to service users of engaging with the scheme in these different ways.

## Method

### Context

On the 17th March 2020, following Government advice issued in relation to the Coronavirus (COVID-19) pandemic, Public Health Wales (PHW) made the decision to suspend delivery of NERS. Those with a minimum of four weeks on the standard face-to-face programme (a quarter of the full 16-week scheme) were asked if they would like to postpone attendance until ‘normal’ service resumed, or to continue receiving support remotely via virtual classes and/or a written home exercise programme (see Table 1 for detail). For those wishing to engage virtually, live and/or pre-recorded exercise sessions were offered (variation per local area). In March 2021, with the easing of some pandemic restrictions, a modified version of the programme started operating, utilising both face-to-face and virtual delivery options. For the modified programme, the mode of delivery offered was based on an assessment, made jointly by the Exercise Referral Professional (ERP) and service user, of the user’s vulnerability to COVID-19 infection and the then current level of infection in the local population (COVID-19 alert level, varying by Welsh district).

**Table 1:** Details of the three programme types delivered by NERS

Programme type	Standard	Remote	Modified
**Timeline**	Scheme initiation (2007)-March 2020	March 2020-March 2021	March 2021 onwards
**Core Elements**	• Referral from a GP or allied health professional • Consultation/first assessment • 16 weeks of exercise sessions (expectation of two sessions per week) • 16-week assessment	As per standard	As per standard
**Location**	In person assessment and exercise sessions at a leisure setting	First consultation in person. Minimum of 4 weeks of face-to-face exercise sessions required to permit starting virtual sessions. Follow-up assessment at 16-weeks held virtually. ‘Check-in’ phone calls made	All elements delivered face-to-face, virtually, or as a mix. Delivery determined for each service user based on a combination of their clinical vulnerability and Welsh national/local Covid-19 alert levels at that time
**Home programme option**	While service users could opt for one of their two weekly exercise sessions to be supported at home by their ERP (using a written home exercise plan), this was not actively encouraged	Service users who did not want virtual delivery could choose to be supported using a written home exercise plan, supplemented with telephone calls with their ERP	As per standard
**Pathways covered**	Generic; stroke; falls prevention; back care; cardiac; pulmonary; cancer; mental health; weight management; lifestyle (for patients awaiting hip/knee replacement); pregnancy	As per standard	As per standard

In March 2021, an evaluation of the impact and opportunities presented by the adaptation to virtual delivery commenced. This followed an expression of interest submitted to the funder (the National Institute for Health and Care Research; NIHR) by PHW and the Welsh Local Government Association (WLGA; WLGA provided operational management of NERS at the time). This was led by PHIRST (Public Health Intervention Responsive Studies Team) Connect, consisting of academics, public contributors, and an independent study advisory board.

### Patient and public involvement and engagement (PPIE)

The evaluation team was supported by a dedicated Public Involvement Research group (PIRg), comprised of members of the public, service users and carers, and also by an independent study advisory board and a NERS-specific advisory group, both of which provided external policy- and practice-orientated advice. Alongside PIRg oversight, a project specific NERS Public Voice (PV) group was convened to provide service user input. This comprised five individuals referred to NERS, four of whom took-up the scheme.

All study forms (e.g. participant information sheet) and interview schedules were reviewed by members of the PIRg and NERS PV group prior to use, including piloting the schedules with two members of NERS PV group. PPIE continued throughout the study, which included the iterative presentation of findings to NERS advisory group, NERS PV group, and the PIRg, all of which contributed insights that informed the analysis. The findings section therefore represents the shared interpretation of researchers, public contributors, and stakeholders.

### Recruitment

Three groups of individuals were targeted for recruitment to this study: practicing NERS Exercise Referral Professionals (ERPs, ‘service providers’); members of the public participating in NERS (‘service users’); and members of the public who did not take up the referral opportunity.

Service providers and service users were recruited with support from the WLGA. Service providers were paid exercise professionals, delivering exercise classes to service users, recruited from among all NERS ERPs then employed on the scheme. This list was provided by the WLGA and contained the names and contact details of all ERPs, along with their employing local authority. Maximum variation sampling was used to obtain service providers that represented as many local authorities as possible. To that end, recruitment proceeded iteratively, with one ERP per local authority invited in turn until either one ERP had consented, or there were no remaining ERPs to invite. Invites were made by email and/or telephone.

Service users were recruited via a contact list supplied by the WLGA containing the details of all individuals enrolled on NERS between January 2019 and November 2021 who consented to have their information shared for research. Beside contact details, information was provided on each individual’s local authority, their assigned NERS pathway, and scheme status: declined remote programme; experienced remote programme; experienced modified programme (see Additional File 4 for full list). As with service provider recruitment, maximum variation sampling was employed. This was used to obtain a sample that represented as many different local authorities, NERS pathways, and programme experiences as possible. As before, recruitment proceeded iteratively to this end. Invites were made by email and/or telephone.

Efforts to recruit people who declined a scheme referral proceeded using a range of methods. Over 20 organisations supporting individuals likely to meet the scheme’s eligibility criteria (e.g. Diabetes UK, British Heart Foundation support groups, Slimming World) were contacted and asked to promote the study via their communication channels (e.g. social media, email). An advert, presented in English and Welsh, was also placed on the NIHR’s People in Research website. Finally, all service users participating in the present study were asked if they knew anyone who had declined a NERS referral.

Service users and those who had declined referral were offered a £10 shopping voucher in return for their time. This was not offered to service providers as focus groups were scheduled to take place during paid working hours.

### Registration, ethical approval, and consent

The protocol for the study is registered on Research Registry (researchregistry7842). Ethics approval was obtained from The University of Hertfordshire’s Health, Science, Engineering and Technology Ethics Committee with Delegated Authority (ECDA; protocol number aLMS/SF/UH/04546(3). Email invites and study adverts contained a link to the study’s project site on REDCap, a secure data capture and management platform, where interested individuals could find out more about the study. If they wished, individuals could then proceed to provide e-consent and basic demographic information before selecting a focus group (service providers) or interview (service users and those who had declined NERS) time slot. Study emails, invites and information on the REDCap project site were presented in both English and Welsh.

### Data collection

Semi-structured schedules were used to guide focus groups and interviews (see additional file 1). Schedules were designed to guide participants to talk through the sequence of activities experienced by NERS users (from initial referral to final consultation). Questions prompted individuals to discuss (and contrast where applicable) experiences of activities delivered in either face-to-face and/or virtual format, and the impact on scheme uptake, engagement, and delivery. Through a short set of structured questions at the end of the interview (see additional file 2), service users were asked about expenses (‘out-of-pocket’ costs) they had incurred while engaging in NERS and whether these impacted on their engagement. These questions asked about expenses incurred through face-to-face and virtual delivery formats, alongside other general costs (e.g. exercise clothing required irrespective of delivery format).

Data were collected between April and December 2021. One-to-one interviews were conducted by a single member of the research team whereas all focus groups were co-facilitated. In two of the three focus groups, co-facilitation was by a researcher and a NERS coordinator trained in qualitative data collection methods. The remaining focus group was co-facilitated by two researchers. All data were collected remotely due to COVID-19 pandemic restrictions on social contact. Focus groups were conducted via Zoom, while interviews were held either via Zoom (*n* = 17), Microsoft Teams (*n* = 1), or by telephone (*n* = 4) as per the participant’s preference. Focus groups lasted between 87 and 107 min, and interviews lasted between 23 and 80 min. All focus groups and interviews were audio recorded and transcribed verbatim.

### Analysis

Qualitative data were analysed using Framework Analysis. This method offers a systematic approach to analysis that can be utilised in research teams with a range of experiences, including public members [27]. Framework analysis is an ‘open, critical and reflexive approach’ consisting of seven stages: transcription; transcript familiarisation; coding; developing an analytical framework; applying the framework; charting data into the framework matrix; interpreting data [26]. NVivo 12 software was used to assist with data management, coding, and indexing. Focus group data were analysed first. Two researchers (KN and NH) independently coded all three transcripts. In the first instance, both researchers coded a single transcript, aiming to identify positive and negative views or experiences of virtual delivery (or, conversely, of face-to-face delivery). They then met to review their developing analytical frameworks. As there was good agreement in identified sub-codes, the remaining transcripts were coded. After coding the final two transcripts, the two researchers met again for consensus discussion. The two frameworks were compared, and decisions made about which codes should be retained (including names and definitions applied) and the text to be indexed against them.

Next, interview data were incorporated into the existing analytical framework. First, five transcripts were independently coded by the same two researchers using the analytical framework developed through the focus group analysis. As before, the researchers met to review coding and agreed some additional codes to extend the framework. A single researcher (KN) then proceeded to code all remaining transcripts using this framework.

Microsoft Excel was used to create a framework matrix for the combined focus group and interview data with illustrative quotes included in the relevant cells of the matrix. One researcher (KN) led data interpretation and identification of themes. During this stage, there were several points at which stakeholders and public involvement groups fed back on provisional themes, enhancing rigour of the analytical process. Quotations have been selected to illustrate themes. In result presentation, codes follow each quotation to indicate the associated participant’s ID, which incorporates their gender identity (female: F; male: M). Information on their participant group (whether service provider, service user, or declined scheme), and where relevant, their programme type (standard, remote, or modified), the mode of delivery received (virtual or face-to-face), and programme completion (completed or withdrew), is provided in additional file 4. The results are organised by major theme and associated sub-themes. Unless otherwise stated, evidence of each sub-theme was present across all versions of programme delivery (standard, remote, or modified).

Data on ‘out-of-pocket’ costs were analysed and summarised by APW using descriptive statistics (see additional file 3 for further detail).

## Results

### Characteristics of participants

In total, 19 service providers participated across three focus groups, and a further 21 service users and one person who declined NERS, took part in an interview. Of the service users interviewed, four declined the remote programme, eleven had chosen to go ahead with the remote programme (nine completed and two withdrew), one withdrew from the standard programme, and five received the modified programme (four exclusively face-to-face delivery, and one exclusively virtual delivery). Twenty local authorities were represented by the participant group. Table 2 below presents the summary characteristics of the sample. See additional file 4 For a more detailed breakdown of participant characteristics.

**Table 2:** Summary characteristics of the sample

	Service providers	Service users	Individual declining referral	Total
	N (%)	N (%)	N (%)	N (%)
**Gender**				
Female	9 (47.4)	12 (57.1)	1 (100.0)	22 (53.7)
Male	10 (52.6)	9 (42.9)	0 (0)	19 (46.3)
**Ethnicity**				
White	18 (94.7)	21 (100.0)	1 (100.0)	40 (97.6)
Prefer not to say	1 (5.3)	0 (0)	0 (0)	1 (2.3)
**Age**				
18–44	11 (57.9)	0	1(100.0)	12 (29.3)
45–59	8 (42.1)	4 (19.0)	0 (0)	12 (29.3)
60+	0	17 (81.0)	0 (0)	17 (41.4)
LA deprivation*				
Low	13 (68.4%)	19 (90.5%)	Unknown	32 (80.0%)
High	6 (31.6%)	2 (9.5%)	Unknown	8 (20.0%)
**LAs represented**	17	13	Unknown	20

*Based on the percentage of Lower Super Output Areas within each local authority which are ranked in the most deprived 50% of LSOAs in Wales (low is below the national average (50%) and high is above). LA = local authority

### Themes

Five themes encompassing 15 sub-themes were generated during data analysis. The five main themes: (1) Opaqueness and uncertainty around referral; (2) ERPs allaying concerns and providing reassurance at scheme entry; (3) Mixed appeal and accessibility of virtual delivery; (4) Factors that support ongoing engagement; and (5) Personal and financial circumstances restricting uptake and engagement. Out-of-pocket (OOP) costs incurred by service users are presented alongside theme five to provide additional context. Each theme follows below with an explanation of the sub-themes it encompasses, alongside illustrative excerpts from participant data.

#### Opaqueness and uncertainty around referral

Referral through primary or secondary care is the point of entry to NERS. Three sub-themes characterise what participants said about this aspect of the service: (i) *Lack of information and poor promotion of NERS*; (ii) *Delay in starting NERS and anxieties about ‘fitting in’;* and (iii) *motivation to improve health and quality of life*. Note, all service users interviewed were expecting to receive face-to-face delivery at time of referral, most of whom went on to receive this, at least initially. This theme therefore exclusively relates to uptake of a face-to-face NERS delivery.

##### Lack of information and poor promotion of NERS

Service users were underwhelmed by the referral process into NERS, particularly through GP practices. Commonly, for example, service users reported that their GP was unable to tell them much about NERS, and that they, not their GP, were the referral instigator:

*Well*,* I don’t think they were quite as proactive as they could have been. I think it was more just a case of*,* well*,* here’s a number*,* give them a ring. That’s it*,* really*,* I suppose*,* and it wasn’t - they weren’t particularly overly trying to make me feel like I should go*,* or anything like that. I… don’t think they really knew huge amount about it*,* to be honest* [F19].

##### Delay in starting NERS and anxieties about ‘fitting in’

Several service users talked about their frustration with the delay between referral and starting on the scheme:

*The only thing with me*,* the actual correspondence was fine*,* and they actually telephoned me with an assessment date*,* but…I waited quite a long time between the GP referral and actually getting an assessment date* [F14].

Service users also recollected anxieties about the scheme during this period. ‘Fitting in’ was the most frequently reported anxiety, reflecting an underlying belief that leisure centres were predominantly frequented by young, fit, and competent exercisers:

*I think gyms can be very… I think a lot of people… they wouldn’t go to the gym*,* because they see the gym as a place for people who know what they’re doing* [F11].

##### Motivation to improve health and quality of life

Despite experiencing barriers to uptake, service users we spoke with had overcome these to enter the scheme. All service users articulated having a clear and explicit reason for requesting or accepting the referral. Most commonly, this was the desire to regain or maintain a level of health or quality of life:

*I felt the radiotherapy that I had sort of zapped every bit of strength from me. So that’s what I really wanted*,* my strength back and health*,* as it were* [F18].

For some, the scheme also presented a reason to get ‘out and about’, providing a focus to their day and opportunities to interact with others:

*What appealed to me was actually getting out and about*,* and being healthier and helping*,* using exercise to help me lose weight as well*,* so yeah* [M11].

#### Exercise referral professionals (ERPs) allaying concerns and proving reassurance at scheme entry

Following referral, service users’ next significant encounter is with the ERP. This experience impacted uptake although, unlike referral, the effect tended to be more positive. Two sub-themes encompass how ERP contact enhanced engagement: (i) *Helping those referred to overcome anxieties about attending;* and (ii) *Striking the right tone*. Note, almost all service users’ early encounters with their ERPs were face-to-face.

##### Helping those referred to overcome anxieties about attending

ERPs used initial strategies such as phone calls, meeting them at the door on their first visit, and close supervision in the early weeks, to support uptake and engagement:

*Well*,* you did feel that you knew*,* because*,* I mean*,* obviously*,* there’s a trepidation not knowing where you’re going*,* because I’d never been to the leisure centre before. So*,* knowing that there was somebody there*,* and they were friendly*,* but it was nice for them to contact you ahead of time* [F22].

For the participant declining NERS referral, the fact the ERP she spoke with did not allay anxieties related to her health condition contributed to her decision to reject the referral:

*I just don’t really think he really knew enough about fibromyalgia*,* chatting to him. He was kind of like saying*,* oh*,* we’ll do this*,* that and the next thing. And I was thinking*,* that’s going to be way too much… He kind of like fobbed me off a bit*,* I felt*,* and I just was like*,* hmm*,* I’m not too sure about this. So*,* yeah*,* I didn’t feel overly confident in the person that I spoke to* [F19].

##### Striking the right tone

In the first consultation, ERPs facilitated engagement by helping those referred to understand the potential benefits for them, setting expectations, and making people feel comfortable. Service users talked about the support they received and how the tone was set from the first interactions:

*The gym instructors were lovely*,* and you were taken into private room and then within five minutes*,* I was quite relaxed and it was all just chatting*,* what you could get out of the programme and what they could do for you. So after that initial consultation*,* I was fine after that* [F15].

#### Mixed appeal and accessibility of virtual delivery

The mixed appeal of virtual delivery is represented by: (i) *Struggling to engage service users in virtual delivery vs. those who tried virtual not wanting to return to face-to-face delivery;* (ii) *Mixed views on impact of virtual for social interaction and relationship building;* (iii) *Consultations most suited to face-to-face delivery;* and (iv) *Virtual delivery widening access for some*. Note, this theme captures the views of service users and providers when virtual delivery was first offered, and the experiences of those engaging with it.

##### Struggling to engage service users in virtual delivery vs. those who tried virtual not wanting to return to face-to-face delivery

Service providers largely reported service users as preferring face-to-face over virtual delivery. Service users frequently opted to postpone their place on NERS when virtual delivery was offered. Some service providers had more success encouraging users to continue on the scheme, but even then, users were more likely to elect for support through written home exercise plans supplemented with telephone calls, than virtual delivery:

*Yeah*,* so definitely agree with that…. a lot of the clients there*,* or the patients there*,* have stressed they would rather wait for face-to-face classes*,* than would remotely* [M3].

Service providers also stressed however, that many of those that did opt for virtual delivery went on to actively prefer this mode over face-to-face:

*They’re actually not coming back*,* not all of them*,* I’d have said 80% of them are not coming back face-to-face and I don’t think it’s because they’re afraid of COVID or anything like that*,* it’s because they’re happy with what they’re doing*,* they’ve got their group of people that they see each week and they’re in the comfort of their own home* [F7].

##### Mixed views on impact of virtual for social interaction and relationship building

One of the reasons that virtual delivery lacked appeal was because it removed what many perceived as a key benefit – the opportunity to interact with others:

*… an awful lot of people who went*,* are lonely people. They are widows or widowers*,* and actually physically going out on a Tuesday morning to go to the gym*,* is going out and meeting people. And that overrides the virtual*,* because doing the virtual for a lonely person is just like turning the television on*,* isn’t it?* [M13]

However, ERPs did give examples of when and how meaningful online relationships had been established:

*… you’ll always have your ones that will be there as soon as you turn on or 20 min before you turn on*,* you just want to make sure that it’s working and they’re all popping in and I’m just bopping around*,* getting my chair in place*,* getting everything set up and they’re all having a chit-chat about everything [M19*].

Service user accounts also supported this. All those persisting with virtual delivery spoke of having developed or maintained meaningful connections:

*It’s very important for me*,* because*,* as I said before*,* for me*,* it helps to combat the social isolation… a lot of us on the programme are living alone*,* so*,* for us*,* seeing people and having a chat to them and then doing the class*,* and having a bit of a chat afterwards and stuff like that. And getting to know each other on the virtually*,* that was a huge bonus* [F14].

##### Consultations most suited to face-to-face delivery

In contrast to the mixed service user experience, service providers typically described a preference for face-to-face consultations over virtual. They felt that this mode of delivery helped build rapport with new service users and enabled them to identify more subtle indicators of health and wellbeing:

*I’ve got to say*,* I was very*,* ‘no*,* online assessment*,* no-no-no’…I think that 90% of your assessment can be seeing the person walk*,* how they walk into the room*,* and you’ve decided as they walk into the room*,* you know what they need and what you’re going to do with them* [F7].

Further, there was recognition that face-to-face delivery facilitated collection of objective data to evidence progress:

*When it’s virtual you’re not getting all the data that you could possibly get*,* so for example their weight or their BMI or their blood pressure*,* you’re not collating that data so you kind of think*,* well I’m just here to give you sort of health advice* [M2].

##### Virtual delivery widening access for some

Service providers experienced virtual delivery widening access for some groups of people. This was further reinforced by service users, particularly those with a caring responsibility, or living with a disability, mental health problem or long-term condition:

*For her it [virtual] was actually better because she could do it and her dependent would be sat in the corner*,* or in the room next door and obviously she felt safer doing that than leaving her in the house for an hour or so* [M5].

Service providers also identified that virtual delivery has the benefit of increasing the variety of specialist instructors and classes available:

*Through Zoom I’ve been able to work with clients from opposite ends of the county*,* that they don’t necessarily have the instructor up there to do a falls class or a Movement for Wellbeing class but they’re able to still attend my sessions*,* so they’ve really-really appreciated that* [F8].

#### Factors that support ongoing engagement with NERS

Service users and ERPs had consistent views about aspects of NERS that facilitated engagement and completion of the scheme. Virtual delivery was perceived as having both a positive and negative impact on some ‘core ingredients’: (i) *Structure and accountability of NERS;* (ii) *Sense of achievement and evidence of progress*; (iii) *Trust in and support of ERPs;* and (iv) *Safety perceptions of virtual vs. in-person delivery.*

##### Structure and accountability of NERS

Service users talked about how they found the structure and accountability of the scheme, whether in-person or virtual, appealing, and that this had acted to sustain their commitment. Having a formalised exercise plan, along with attendance monitoring, were both key factors:

*There is a target. If you’ve got to go on a Tuesday at a certain time*,* you’ve got a target. You’ve got a target and you’ve got to meet that target. Whereas if it’s on the virtual*,* you don’t even have to open your laptop*,* it’s that easy to avoid it* [M13].

Structure and accountability were equally important however to those opting for virtual delivery:

*It’s the commitment that is important. Without commitment to a specific time*,* a specific day*,* we probably wouldn’t be exercising at all. We’d probably just go back into our old ways* [M14].

Live, as opposed to pre-recorded, virtual sessions were identified as necessary for creating the required accountability.

##### Sense of achievement and evidence of progress

Service users spoke of the sense of achievement they felt as a result of engaging in the scheme, regardless of mode of delivery. Sessions were also experienced as enjoyable and energising:

*I enjoyed those sessions from the first one. Yeah*,* and I did think it made you feel better about yourself*,* and you were actually doing something*,* instead of sitting about* [F22].

Another aspect of the scheme that supported service users’ continued engagement was evidence of making progress, particularly related to health benefits:

*Well*,* I liked the fact that I was actually beginning to feel fitter. That was amazing!… I felt it was doing me good. And it’s like with any exercise that you do*,* you get into a routine of doing something and it becomes easier to do it*,* and you see the benefits of doing it* [F11].

ERP feedback provided tangible evidence of that success:

*He would alter the exercises*,* and give me a variety of different things to do. And he would also note down where I’d improved and he would say to me*,* oh*,* you’ve done more this week than you did last week*,* or you’ve gone up a level* [F21].

There was mixed evidence about whether virtual delivery reduced or increased the speed of progress. Some service users reported putting in less effort due to a perception that they were more ‘hidden’ during virtual delivery:

*I have to say probably the face-to-face was more effective… Now*,* although you’re probably doing the same number of exercises*,* over the same time*,* it’s easier to take it easy if you’re online*,* so you maybe don’t put quite so much effort into the exercises* [M14].

However, one ERP presented a contrasting view of greater effort being expended during virtual delivery and provided the explanation:

*They’ve all said we are most definitely working harder on Zoom… they do talk a lot in the classes… but on Zoom they are just focused on you*,* they can’t speak to anybody else* [F1].

##### Trust in and support of ERPs

Support from and trust in ERPs (regardless of delivery mode) was also central to service users’ ongoing engagement. Service users’ trust and confidence in their ERP, particularly relating to what they could do safely, gave them the confidence to exercise:

*Not just supportive*,* obviously*,* giving me the proper professional guidance as well to make sure I didn’t injure myself*,* or cause any other sort of harm* [M12].

Service users also found words of encouragement particularly motivating. There was a level of service provider skill evident in allowing individuals to be self-directing, whilst also encouraging them to push themselves within safe limits:

*But she’s not demanding that you do*,* and it’s left up to you. So she encourages you to progress*,* if you like*,* but not*,* you’re not demanding in any way that you do do it. So it’s just at the right level*,* really* [F22].

##### Safety perceptions of virtual vs. face-to-face

Service providers had concerns about their impaired ability to assess and respond to visual information online and the related impact of this on users’ safety:

*If you’re doing a lunge for example*,* all you see is chest high*,* you don’t see the internal rotation or splayed foot or whatever it is*,* whereas face-to-face you can see everything and can pinpoint it and put it right straightaway* [M3].

Echoing ERPs, service users also had concerns, particularly those that declined the remote offer or withdrew from virtual delivery:

*And you could actually be just missing a key point about maybe the way your feet are positioned*,* for example*,* and then that’s then detrimental to what you’re supposed to be doing. Whereas if you’ve had the face-to-face person*,* and the person can physically see your feet and say*,* actually*,* you need to just do this with them.* [F11]

Service users also commented on how virtual delivery made it difficult to ask for feedback during sessions, raising further safety concerns. Service providers were particularly concerned about service users falling at home during online classes:

*It feels a little bit pressure on you because if anything does happen*,* if you’re face-to-face there*,* you’re straight on scene*,* you can call a first aider…. everything’s on hand*,* whereas when it’s virtual*,* no…. There’s definitely a little bit more to think about*,* and a bit more complicated* [M2].

To alleviate this, some initially adapted session content. They recognised, however, that this could potentially reduce benefits to service users:

*They’re at home*,* perhaps on their own and so you keep it well within their comfort zone rather than make it perhaps just that little bit more challenging which they’d benefit slightly more [from]* [M9].

#### Personal and financial circumstances restricting uptake and engagement for some

Personal and financial circumstances that might restrict service user uptake and engagement are represented by: (i) *Absorbing session fees and travel costs;* and (ii) *Digital exclusion*. Out-of-pocket costs are included throughout to support this theme’s narrative.

##### Absorbing session fees and travel costs

Out-of-pocket cost analysis highlighted the differing costs service users incurred between engaging with face-to-face and virtual delivery (see additional file 3 for detail). The one universal cost was for exercise clothing and footwear with a mean cost of £29.17 and £25.00 respectively. Face-to-face sessions incurred a mean cost of £2.27 per service user (based on data provided by 16 service users who reported paying on a per-session basis). Further, most (17/20) of those who attended face-to-face sessions travelled to the exercise venue by car, covering an average distance of 3.7 miles per one-way journey, estimated as costing approximately £0.55 (primarily for fuel) or £1.72 (additionally including costs beyond fuel). Only one service user reported that out-of-pocket costs related to face-to-face attendance were problematic:

*With me*,* money is very*,* very tight. I think a lot of us on the programme don’t actually work*,* because of our health conditions and things*,* so money is very tight. I think that even if you did two classes a week*,* that’s two*,* four… Well*,* say*,* there was four weeks in a month*,* that if you did it twice a week*,* that’s what*,* £16 a month…Yeah*,* I would probably have to amend [attend] the classes*,* based on what I think I could afford* [F14].

The individual who declined NERS reported cost as their primary reason for not taking up referral:

*It probably would have been a bit more useful*,* I guess*,* if she’d [GP] understood a bit more about how… Well*,* especially like the money side of things*,* because I was expecting not to have to pay. And*,* actually*,* whereas that would be fine now*,* in that situation*,* I had to recently give up my job*,* because of my caring responsibilities for my child with the disability. And we had quite a lot of money worries at the time* [F19].

It is likely that further evidence of prohibitive costs would have been identified had more people who declined a referral to NERS been recruited.

##### Digital exclusion

Consistent with NERS policy, all attending virtual classes (*n* = 10) reported they had not paid a session fee. There were however some direct costs relating to virtual engagement specifically. Four service users reported buying exercise equipment to engage with virtual sessions at a mean cost of £10.00. Most service users engaging with virtual classes accessed them using broadband at a mean cost per month of £42.00. Two service users commented that broadband costs were not driven by engagement with NERS (e.g. they had purchased broadband for other reasons). One interviewee reported accessing sessions via mobile connectivity with a monthly cost of £19.00. Only one service user reported having to buy a device to access virtual classes.

Accessibility issues related to internet connectivity, access to suitable devices, and having the knowledge and confidence required to access the platforms for virtual sessions, were all barriers to this form of NERS:

*Yeah*,* what I find quite frustrating to be honest*,* mainly for the client more than myself*,* is if they’ve got a bad connection*,* you get on and once you start*,* they freeze*,* and like could be frozen for about five minutes* [M6].

Service users having old or outdated devices was also raised as limiting accessibility:

*But my problem is I’m talking to you from a desktop*,* not a laptop*,* or I’ve got a small tablet*,* but it’s so ancient… I think if I had a very up-to-date tablet that I could have moved into a different room*,* and with a reliable internet connection*,* then I think things may have been different* [M17].

It was evident that in some cases this had led to service users having to obtain new devices to enable participation:

*And I said*,* well*,* I can’t even afford a second-hand at the present time…. So what she [sister] said was that she would buy it for me*,* and then when I had a bit of money*,* I could start paying her off in instalments. Well*,* I haven’t started paying her off yet*,* because I haven’t had no money* [F14].

A related issue concerns the reluctance of some users to engage with virtual delivery due to being unfamiliar with, or lacking confidence in, accessing content online. There was a tendency for this to be most evident among older service users:

*One of the reasons why I don’t like going on that thing*,* and I’m looking at it now*,* is that I don’t really know how it works*,* so… it’s an enemy*,* and not a friend* [M16].

For some service users however, this was a barrier that could be overcome with support from ERPs, family members or friends. Service providers also talked about having to overcome initial problems and anxieties:

*Absolutely*,* we did*,* well I did [have teething problems]*,* technology is not my thing*,* but I quite enjoy them now* [F6].

## Discussion

Our study aimed to understand the implications that delivering NERS in a virtual format may have for scheme uptake, engagement, and delivery. Views and experiences of three groups of stakeholders were explored and contrasted: service providers, service users, and one person who declined referral. Themes identified were: (1) Opaqueness and uncertainty around referral; (2) ERPs allaying concerns and providing reassurance at scheme entry; (3) Mixed accessibility and appeal of virtual delivery; (4) Factors that support ongoing engagement; and (5) Personal and financial circumstances restricting uptake and engagement.

Reflecting previous research on stakeholder perceptions and experiences of NERS referral [28], service users largely found their referrer lacked knowledge about the scheme, what it entailed, and how to make a referral. Service users’ openness to the scheme at referral was linked to their desire to achieve valued health and quality of life goals. For some, this motivation is likely to have been initiated by a health scare, illness, or medical treatment. Significant life-events such as these can be particularly effective in starting behaviour change, especially if prior behaviour is perceived to be cause of the crisis [29]. This raises the question of whether a threshold level of motivation and health literacy might be required of individuals to be able to access the scheme. Of concern, this may create inequalities in uptake whereby individuals’ characteristics and personal circumstances dictate whether they stand to benefit from it [30, 31]. As NERS gatekeepers, it is important that referrers are equipped to have brief opportunistic conversations with eligible patients that develop their motivation to act. Patients at risk of future illness, but yet to experience a significant health event, may in particular require this.

Echoing other research on barriers to accessing exercise referral schemes (20,21), service users held anxieties relating to the leisure centre environment and not fitting in. In all cases, however, these anxieties were overcome, sometimes helped by their ERP who offered support and reassurance – such as through an introductory phone call. This raises the possibility that, alongside individuals being lost at referral, others are lost in this intervening period during which anxieties emerge but are not allayed. However, our study was unable to recruit sufficient individuals declining NERS to substantiate this.

Research examining the patterned uptake of NERS [19] indicates that certain groups are less likely to take up referral than others, such as those from more deprived areas. Wider evidence also indicates that people are more likely to initiate behaviour change when their physical and psychological resources allow for this [32]. Despite being highly motivated to join the scheme to manage symptoms, the one decliner interviewed reluctantly turned down the scheme due to cost, and another reported session and travel costs being limiting factors. These findings reflect existing research that has identified location and cost factors as barriers to uptake and adherence with face-to-face exercise referral schemes (21,23). Our study also found that costs may present a barrier to engagement, with virtual delivery for one service user necessitating borrowing money to obtain a laptop for virtual engagement. The sample in this study is biased towards those for whom cost is not prohibitive of attendance; all service users interviewed were by their very nature those who were able to engage. Research to explore the factors underlying decisions to decline referral, or failure to start, or to withdraw, are needed to better understand how these factors can be addressed to ensure equitable access to NERS.

Reactions of service users to the offer of virtual delivery provide an indication of material and psychological barriers to accessing digital content. Lack of access to a suitable device, absence of a home internet connection, or having a weak/unstable connection, were all identified as preventing access to virtual delivery, or impairing experience resulting in disengagement. Further, it was clear that some users lacked digital skills or confidence to get online and utilise the necessary platforms. This was most common amongst older service users. NERS intakes typically feature a high proportion of older adults, as reflected in the present study where over 40% of participants were 60 + years. Given evidence that over 75% of UK internet non-users are aged 65 years or above [26], digital literacy levels are likely to be an ongoing barrier to virtual delivery for this cohort. Some older service users who initially struggled to get online, went on to embrace virtual delivery once supported to do so. If NERS is to continue virtual delivery, service users’ lack of skill or confidence online should be met with support to overcome these barriers, perhaps offering guides or signposting schemes that support digital literacy.

There were others however whose circumstances meant that accessibility issues were insurmountable. Financial barriers along with poor connectivity, prevented others from engaging. The importance of digital inclusion has been recognised by the Welsh government, with older people, those living with disability, and those who are unemployed or economically inactive (e.g. retired, too ill to work) more likely to experience exclusion [33]. The present study indicates that inequalities in scheme uptake could be reduced if digital accessibility is addressed. Of importance, there is overlap in the groups at greatest risk of digital exclusion, and those facing the greatest barriers to accessing NERS when delivered face-to-face. Providing arrangements are made to address digital access needs, a virtual version of NERS may serve to increase uptake and engagement among those who are currently underserved and who stand to benefit the most from participation.

This study identified several aspects of scheme delivery that promoted ongoing engagement, including scheduled classes, with attendance monitored by ERPs. Praise from ERPs for effort and progress made was also perceived as reinforcing. It is important that the focus of NERS is on developing exercise routines that can be maintained beyond the active intervention period, with the formation of habits a key mechanism for achieving this [33, 34]. Repeatedly performing an action in a stable context and experiencing reward for performance aids the development of habitual behaviour [35]. Sessions at scheduled timepoints, with attendance recorded and effort praised can only really be achieved through live sessions (as opposed to pre-recorded).

Experiencing reward in the first few weeks of the scheme is likely to be particularly important. This time is often characterised by apprehension and anxiety relating to the new environment and behaviour. ERP approval and praise, whilst initially important, needs to be gradually replaced by intrinsic reward that persists beyond the end of the active intervention. This was evident in the present study, where service users experienced exercise or its immediate outcomes as enjoyable, or as having a stress reducing effect. The experience of intrinsic reward has been associated with habit formation [36, 37] and adherence to exercise referral schemes (22). Regardless of the mode of delivery, it is encouraging that NERS can provide the opportunity for people to experience intrinsic rewards from exercise which can provide exercise reinforcement beyond the end of the scheme [38–40].

A further predictor of long-term behavioural engagement is satisfaction with experienced outcomes [41–43]. Satisfaction, also conceptualised as a type of intrinsic reward, serves both to indicate to the individual that their initial decision to adopt a new behaviour was correct, and to provide a continued source of motivation to engage in behaviour long-term [43]. In the present study, service users experienced their own progress through for example, improved flexibility, stamina, strength, or relief from illness symptoms, all of which motivated continued effort. ERPs were found to reinforce these beliefs by demonstrating that they too had observed changes, or through giving feedback on objective measures at scheme check-ins. Positive feedback such as this has been shown to support continuation of a new behaviour by promoting autonomy and competence [44], and consequently intrinsic interest [45].

There was some evidence that virtual delivery potentially limited progress. Although one ERP reported service users working harder in virtual classes, service users reported that they sometimes put in less effort due to the perception that they were more ‘hidden’. Further, ERPs reported concerns that the virtual environment could elevate the risk of falls or injury, leading them to make adaptations which may have reduced efficacy, such as changing standing routines to seated ones. Given the potential importance of service users experiencing satisfaction with outcomes, NERS should take steps to ensure that benefits are not diluted for virtual delivery. Safety concerns are less easily overcome but given that those who are living with illness and disability are more likely to benefit from virtual delivery, this is worth careful thought. It could be for example, that those who have demonstrated competence by safely performing exercises in a face-to-face environment could graduate to joining classes virtually from home, where this is a preference.

Service users’ concern that exercise may worsen an existing injury or illness was a potential barrier. It follows therefore that trust in ERPs to protect their health and wellbeing whilst exercising was a facilitator of engagement, and further, that safety concerns were a barrier to uptake of virtual delivery. Exercise self-efficacy, the belief that one can successfully engage in a behaviour, has been identified as a key determinant of physical activity [46–49]. If an individual does not believe that they can exercise without injury, then self-efficacy will likely be too low for behaviour to be initiated and repeated. ERPs likely serve as an important source of self-efficacy through their demonstration of how to perform exercises correctly, and then giving feedback and direction to users to ensure good form [50]. It may be that the virtual environment threatens exercise self-efficacy particularly where this introduces risk outside of ERP control. Given the central importance of safety to NERS service users, uptake may be facilitated through highlighting the specialist, condition-specific knowledge that ERPs have at referral. Further, if virtual delivery of the scheme is continued, then measures that have already been taken to enhance safety, such as having a supernumerary ERP at each session to monitor safety, should be promoted.

Virtual delivery was perceived as limiting social interactions and was frequently cited as a reason for declining virtual delivery or withdrawing. Many theories of behaviour change emphasise the importance of a supportive social environment for behavioural initiation and maintenance [32]. Further, social support has previously been identified as a facilitator of adherence to NERS [18] and other exercise referral schemes (21–23). Positive social influences facilitate behaviour change by lowering the effort required to initiate and maintain new behaviour through help or encouragement. What is clear, is that it is possible to support service users to develop meaningful relationships online through providing time to socialise during virtual classes. This opportunity developed organically during the pandemic, initially as a result of ERPs allowing time at the start of sessions to resolve technical issues. There are also other potential opportunities to boost this through for example, providing a dedicated online space for peers to share experiences and provide support. If service users wishing to access virtual delivery are required to ‘graduate’ from face-to-face delivery as suggested above, this may have the added benefit of enabling social connections to be developed prior to moving sessions online.

### Strengths and limitations

This study provided an in-depth understanding of the implications of delivering NERS virtually. It identified potential opportunities afforded by this new mode of delivery and considerations that should be made to ensure equivalent levels of user experience and outcomes. All research materials had public involvement in their development and piloting. Reliability, trustworthiness, and breadth of interpretation was increased by the involvement of multiple researchers, members of the public, and stakeholders in the analysis. Data from different stakeholder groups enabled experiences of NERS to be examined from alternative perspectives, consequently providing a fuller account of the phenomena. Further, recruitment methods employed enabled a good cross-section of local authorities to be represented, along with different scheme pathways, and modes of delivery experienced by service users.

However, the study should be interpreted within the context of its limitations. First, most service users interviewed were aged over 60 providing a sample that was skewed towards older adults. Nonetheless, this is consistent with the average age of 56 years from a cohort of nearly 29,000 NERS service users analysed in a related study [51]. Second, despite good representation of local authorities, only a small proportion of these are classed as high deprivation, and the service user group exclusively identified as having White ethnicity. Existing data indicates that those from the most deprived groups are least likely to take up the scheme (18,19). Given this, it is essential that underserved and more diverse populations are heard from if uptake and engagement are to be improved. Specifically, future research should include representation from those living in areas of higher deprivation and a range of ethnic backgrounds so that potential barriers to scheme uptake, including cost, can be explored more deeply.

Despite multiple approaches to recruiting individuals who declined NERS being used, only one such participant was recruited. A reliance on digital methods of advertising the study, as well as having no direct access to individuals who declined NERS, likely contributed. Future research must address this through employing alternative recruitment methods. For this to be successful, sufficient time and resource allocations need to be made to enable researchers to build networks and trust on the ground within relevant communities.

## Conclusions

The present study indicates that offering a virtual version of NERS alongside the face-to-face version could make the scheme more accessible to those who are typically underserved, providing strategies to address digital exclusion are addressed. Key ingredients of NERS which are likely to promote engagement and the formation of long-term exercise habits, are largely undisturbed by virtual delivery. Caveats to this include that the virtual classes should be in a live format. Consideration must also be given to how to deliver classes that challenge the service user, such that meaningful outcomes are achieved, whilst also ensuring safety. The present study also indicates that consultations at which objective baseline and follow-up measurements of health and fitness are taken are most suited to face-to-face delivery. These findings provide evidence that can be used to inform decision-making about the future implementation of virtual delivery within NERS as well as for other exercise referral schemes and wider public health services.

## Electronic supplementary material

Below is the link to the electronic supplementary material.

Supplementary Material 1

Supplementary Material 2

Supplementary Material 3

Supplementary Material 4

## Data Availability

Anonymised copies of the interview and focus group transcripts, along with a thematic aggregation of excerpted text (framework matrix), are available via the University of Hertfordshire’s Research Archive 10.18745/ds.26105.
