# Supplementary material for: Adaptations to the welsh national exercise referral scheme during the COVID-19 pandemic: a qualitative study exploring the experiences of service users and providers and supplementary out-of-pocket cost analysis
Source: BMC Public Health. 2025 Feb 1;25:406. doi: 10.1186/s12889-025-21502-3 (PMC11786398; doi:10.1186/s12889-025-21502-3)
Supplement: Supplementary file 1 — Supplementary Material 1 [file 12889_2025_21502_MOESM1_ESM.docx]

**Details of out-of-pocket costs reported by participants**

| **Domain** | **Variable** | **Sample size (N)** | **n/Mean** | **SD** | **Min** | **Max** |
| --- | --- | --- | --- | --- | --- | --- |
| Face-to-face engagement costs  (20 people attended this  session type) | Charge (£) | 20 | 1.81 | 1.44 | 0.00 | 6.00 |
|  | Charge among those reporting a fee (£) | 16 | 2.27 | 1.24 | 0.20 | 6.00 |
|  | Travel: car (n) | 20 | 17 |  |  |  |
|  | Travel: bus (n) | 20 | 1 |  |  |  |
|  | Travel: taxi (n) | 20 | 1 |  |  |  |
|  | Travel: foot (n) | 20 | 1 |  |  |  |
|  | Car: distance (miles) | 17 | 3.66 | 3.04 | 0.50 | 10.00 |
|  | Car: cost (HMRC, £) | 17 | 0.55 | 0.46 | 0.07 | 1.50 |
|  | Car: cost (NimbleFin, £) | 17 | 1.72 | 1.43 | 0.23 | 4.70 |
|  | Car: cost parking (£) | 17 | 0.00 | 0.00 | 0.00 | 0.00 |
|  | Bus: cost (£) | 1 | 0.00 | - | 0.00 | 0.00 |
|  | Taxi: cost (£) | 1 | 4.50 | - | 4.50 | 4.50 |
| Virtual engagement costs  (10 people attended  this session type) | Charge (£) | 10 | 0.00 | 0.00 | 0.00 | 0.00 |
|  | Equipment purchased (n) | 10 | 4 |  |  |  |
|  | Equipment cost (£) | 3 | 10.00 | 0.00 | 10.00 | 10.00 |
|  | Internet: broadband (n) | 9 | 8 |  |  |  |
|  | Internet: broadband - cost (£) | 7 | 30.00 | 25.33 | 0.00 | 70.00 |
|  | Internet: broadband - cost among those reporting a cost (£) | 5 | 42.00 | 18.23 | 25.00 | 70.00 |
|  | Internet: mobile - cost (£) | 1 | 19.00 | - | 19.00 | 19.00 |
| Other costs | Clothing purchased (n) | 21 | 6 |  |  |  |
|  | Clothing costs (£) | 6 | 29.17 | 13.20 | 15.00 | 50.00 |
|  | Footwear purchased (n) | 21 | 2 |  |  |  |
|  | Footwear costs (£) | 2 | 25.00 | 7.07 | 20.00 | 30.00 |
| Impact | Costs impacted NERS use: Yes (n) | 20 | 1 |  |  |  |

A hyphen indicates a value is not calculable (e.g. a standard deviation (SD) from a single observation). Sample size varies depending on the relevant group and the presence of missing data.

**Table notes**

All expenses are reported in pound sterling (£). It was not feasible to collect details on when costs were incurred, thus no adjustment has been made for the impact of inflation. Given that most costs occurred before the steep rise in inflation during 2022, adjustments would have had relatively little impact on *within* dataset comparisons as the rate of inflation before this time was low. Interviewees varied in how long they had been with the service (see additional file 4), ranging approximately from four weeks to nearly seven years: thus, face-to-face and general out-of-pocket expenses could have occurred at any point during this period, while virtual delivery related costs should relate to more recent expenditure (i.e. from March 2020, when the NERS began virtual delivery).

Some data cleaning was required prior to analysis. Where an interviewee reported a range of values (e.g. a cost of ‘£10-£15’) a mid-point value was used for this analysis.

All reported travel costs are for a ‘single’ one-way journey (e.g. travel *to* a NERS session). To estimate car travel costs, reported travel distance (miles) was multiplied by a cost-per-mile. Two different rates were used:

- HMRC (HMRC 2022) advisory rate which is used to ‘reimburse employees for business travel in their company cars.’ We use the rate of 15 pence per mile, based on a petrol car with an engine size of 1401-2000cc.
- A rate from the ‘NimbleFins’ website which calculates the average cost of owning a car in the UK and divides this by the average travel distance to get a cost of 47 pence per mile (Yurday 2022). This rate is higher partly because it represents a broader ‘cost’, capturing other elements of car ownership beyond fuel (e.g. insurance).

**Narrative summary of findings**

Among those 20 attending F2F sessions, 16 were charged a fee per session, with a mean cost of £2.27, ranging from £0.2 to £6. Four people were not charged directly, with three reporting their attendance was funded via a gym/leisure centre membership (£15-£20 per month). Most (17/20) travelled to the exercise venue by car, covering an average distance of 3.66 miles per one-way journey, estimated as costing approximately £0.55 (HMRC rate) or £1.72 (NimbleFin rate). Only one person travelled by bus (at no cost, as they had a free bus pass) and one by taxi (cost=£4.5).

Among those ten attending virtual sessions, none reported paying a session fee. Four reported buying exercise equipment to engage with virtual sessions, with three reporting a mean cost of £10. Among the nine answering, the majority (n=8) accessed virtual sessions using broadband. Five of these reported a mean broadband cost per month of £42. Two people did not report a broadband cost, as they considered broadband costs to not be driven by engaging with the NERS (which may apply to the other interviewees reporting this cost). One participant reported accessing sessions via mobile connectivity with a monthly cost of £19.

Across the 21 interviewees, six reported buying clothing and two reported buying footwear to engage with the NERS, with mean costs of £29.17 and £25 respectively.

One person noted that these OOP expenses affected their engagement with the NERS, noting that they only do as `many classes as could afford’ at £2 per session. This person attended both types of session, travelled by foot when attending F2F sessions, reported spending £10 on equipment for virtual sessions and no further costs. In qualitative comments, three people explicitly commented that they considered the F2F charges reasonable.

**References**

HMRC. 2022. “Advisory fuel rates.” Internet. <https://www.gov.uk/guidance/advisory-fuel-rates> [Accessed 30.03.2022]

Yurday, Erin. 2022. “Average Cost to Run a Car UK 2022.” Internet. <https://www.nimblefins.co.uk/cheap-car-insurance/average-cost-run-car-uk> [Accessed 30.03.2022]
