# Supplementary material for: Adaptations to the welsh national exercise referral scheme during the COVID-19 pandemic: a qualitative study exploring the experiences of service users and providers and supplementary out-of-pocket cost analysis
Source: BMC Public Health. 2025 Feb 1;25:406. doi: 10.1186/s12889-025-21502-3 (PMC11786398; doi:10.1186/s12889-025-21502-3)
Supplement: Supplementary file 2 — Supplementary Material 2 [file 12889_2025_21502_MOESM2_ESM.pdf]

Costs incurred

Record ID ______________________

Introductory text:
We would like to understand any costs that service users must pay for themselves to participate in the NERS programme

Approximately how many weeks of NERS exercise sessions did they have in total? ______________________

What type of sessions did they have?
- ◯ face-to-face only
- ◯ virtual only
- ◯ Both

On average how many of these sessions did they attend per week? ______________________
(Approx value fine)

For approximately how many of these [ners_total_weeks] weeks were the sessions face-to-face? ______________________

During a typical week when they were receiving face-to-face delivery, how many sessions on average were they attending? ______________________
(Approx value fine)

For approximately how many of these [ners_total_weeks] weeks were the sessions virtual? ______________________

During a typical week when they were receiving virtual delivery, how many sessions on average were they attending? ______________________
(Approx value fine)

What did they pay for the face-to-face exercise sessions?
[note whether this was per sessions, per month etc] ______________________

What (if anything) did they pay for the virtual exercise sessions/home programme?
[note whether this was per sessions, per month etc; if nothing enter '0'] ______________________

What was their main form of travel to their face-to-face exercise sessions?
- ◯ car
- ◯ bus
- ◯ taxi
- ◯ train
- ◯ on foot
- ◯ bike
- ◯ other

Approx how many miles is it from their home to the venue (i.e. one-way only)?
[Explain that asking this so that can work out cost of travelling by car for them] ______________________
(If they don't know, ask them name of home town and where venue located so that we can calculate)

How much did they pay for parking (if anything)?<br>[if nothing, enter '0']	__________________________________
What did they pay for a bus ticket?	__________________________________
Is this the cost given for a single or return bus ticket?	◯ single<br>◯ return
How much did the taxi cost (one way)?	__________________________________
How much is a train ticket?	__________________________________
Is this the cost given for a single or return train ticket?	◯ single<br>◯ return
Were there any costs associated with this form of travel?<br>[If so, detail here (make sure to note whether figure given is for a single or return trip]	__________________________________
Did they buy any clothing (e.g. gym clothes, leggings etc) especially for the NERS exercise sessions?<br><br>[regardless of whether for face-to-face of virtual delivery]	◯ Yes<br>◯ No
How much did they spend on clothing?	__________________________________<br>(Approx value fine)
Did they buy any footwear (e.g. trainers) especially for the NERS exercise sessions?<br><br>[regardless of whether for face-to-face of virtual delivery]	◯ Yes<br>◯ No
How much did they spend on footwear?	__________________________________<br>(Approx value fine)
Did they have to buy any exercise equipment (e.g. gym mat, weights) to enable them to exercise from home?	◯ Yes<br>◯ No
How much did they spend on equipment?	__________________________________
Did they have to buy and devices etc (e.g. laptop, tablet, smart tv, cables) so that they could take part in the virtual exercise sessions?	◯ Yes<br>◯ No
How much did they spend on devices etc?	__________________________________

What was the main way they connected to the virtual content?	◯ Home broadband/fibre<br>◯ Through 4G or 5G signal on phone or tablet
How much per month were they paying for their home broadband/fibre at that time?<br>[if they don't know, ask if know who provider is/was and whether broadband or fibre and then we will try and find out]	__________________________________<br>(Approx value fine)
How much per month were they paying for 4G/5G at that time<br>[if they don't know, ask if know who provider is/was and we will try and find out)	__________________________________<br>(Approx value fine)
Did these costs impact on you being able to use/engage with NERS?	◯ Yes<br>◯ No
Space to note any further comments from participant on costs	____
