# Supplementary material for: Adaptations to the welsh national exercise referral scheme during the COVID-19 pandemic: a qualitative study exploring the experiences of service users and providers and supplementary out-of-pocket cost analysis
Source: BMC Public Health. 2025 Feb 1;25:406. doi: 10.1186/s12889-025-21502-3 (PMC11786398; doi:10.1186/s12889-025-21502-3)
Supplement: Supplementary file 3 — Supplementary Material 3 [file 12889_2025_21502_MOESM3_ESM.docx]

**[SERIVCE PROVIDER FOCUS GROUP SCHEDULE]**


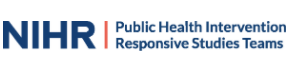


NOTE – This semi-structured schedule covers the topics that will be included in the focus groups, but **the exact wording and order will vary depending on the responses**. Later focus groups are likely to include additional areas which arise from analysis of the first set of transcripts. Sensitive use of prompts is likely to encourage participants to expand their opinions on any of the topics in the schedule, looking particularly for anything that might have a story attached to it.

**Introduction and ground rules**

The researcher will confirm participants’ names and that they have a stable connection and are ready to take part in the focus group. The researcher will again confirm their consent and describe the ground rules:

- Participants should treat the content of their discussion as confidential. As such we ask that they do not share or disclose anything mentioned here with others. At the same time, we cannot enforce this and suggest that they only share as much as they are comfortable doing
- Participants will have the right to pause their participation, withdraw from participating, or avoid talking about any issue they find sensitive. They will not have to provide an explanation for this
- Participants will be informed that this is a ‘safe space’ meaning that all and any opinions are welcome so long as there is respect among participants. Participants can raise a ‘virtual hand’ or physical one on camera to request speaking (this may not be necessary in smaller groups)
- Participants will be requested to abide by the Chatham House rule, and not disclose outside the focus group, any specific details discussed within the group, or what any particular participant has said

The researcher will introduce the goal of the focus groups and the schedule for the meeting.

Positioning of the focus group:

- Those who commission/manage NERS want to understand what can be learnt about adaptations made in response to COVID, particularly in relation to the change from face-to-face to remote delivery
- They are aware that this will likely have had both positive and negative effects for the people that you support through the programme
- They are interested in continuing to offer aspects of the programme remotely in the long-term but want to understand who you think this would/wouldn’t suit, and if there are aspects of the programme that you think should always be face-to-face as a preference
- They are also aware of the significant shift in working style/patterns that this brought for you and the amount of work that has gone into enabling the programme to continue
- They want to understand more about this and how you can be best supported to continue offering alternative forms of delivery in the future

*Before you start, establish what term they use to describe the people they support through the programme: participants, service users, clients etc. Identify/agree the term to be used and replace that with the term ‘service user’ which is used in italics throughout the rest of this schedule.*

*Also, explain that you will be using the term ‘remote’ throughout and that by that you mean anything they have been doing over the past 12 months or so in order to continue to support service users which avoided face-to-face contact e.g. live sessions, recorded sessions, walking challenges etc.*

Firstly. We want to understand the effect that offering a new style of programme, one in which there are some remote elements of delivery may have on programme **uptake**

Whilst we will be speaking to *service users* ourselves, and also people who were referred on to the programme but didn’t take up the offer, we would like to know if you have any views on this?

Your perspectives may come from your overall knowledge/experience with NERS *service users*, or specific conversations you’ve had or observations that you have made during the past 12 months’ or so.

- When the NERS programme is first discussed with a potential service user (i.e. at referral), who do you think would find the inclusion of remote aspects of delivery most appealing (e.g. age group, health needs)? Why?
- Similarly, who do you think would be put off by this? Why? What kind of concerns do you think they’d have?
- One concern with any type of public health programme is that they don’t attract people who need them the most. Do you have a view on the types of people who are least likely to come to the NERS programme? Are these the same people who will be put off by remote delivery i.e. could it compound the problem?
- Do you have any thoughts on what support could/should be offered to those who are least likely to take up the referral? What could/should be said at the referral or in the invite letter to help people overcome any concerns (including of remote delivery)? How can they best be supported/encouraged to get to the point of attending the first meeting?

When the first wave of COVID hit the NERS programme was temporarily paused, after which *service users* on the programme were contacted and asked if they would like to postpone attendance until face-to-face returned, or to continue in a remote format.

- Did you notice any differences between the people who postponed and those who continued? What were these?
- What were people’s reasons for postponing? And their concerns about continuing?
- Were there people who required encouragement/assistance to continue in this format? What did you say/do to encourage/assist them?

**Engagement**

We would now like to ask you about the initial consultation/assessment and the 16-week assessment

- What are your thoughts on conducting the first consultation/assessment remotely? What about the 16-week assessment? Does it work? What is this experience like for you/ for them?
- What is gained from doing it remotely? What is lost?
- Particularly in relation to the first assessment, does running it remotely have an impact on your ability to build trust/rapport? Why? Is this with everyone or just some people? Who?
- What does or could make running the first consultation or the 16-week assessment more difficult/easier?
- Are there any groups of *service users* that the remote delivery of these sessions is more or less suitable for? Why? Are there individuals who should always have this face-to-face where possible? Who/why?
- Do you have a preference as to whether this element of the programme is delivered remotely or face-to-face? Why? [*explore reasons relating to service users and to them*]

I would now like to focus on the 16-week programme of exercise sessions and support.

- Please can you tell me what new things (remote delivery-wise) you have been doing over the last 12 months? [*explore exercise and support e.g. virtual coffee mornings*]
- Did you have any teething problems with remote delivery at the start? Did you feel confident with this new format? What about now?
- Can you walk me through the preparation work that goes into remote delivery? What differences are there to face-to-face preparation?
- Are there any groups of people who in general need more support from you? Who/why? What type of support? Are there any groups that need less input from you than others? Who/why?
- How well have your *service users* responded to remote delivery/support?
- What are your views on who remote sessions/support work for best (age, programme pathway, specific health needs etc)? Why is this?
- And what about who they work for least? Who needs most support to engage and what sort of support is this? Are there any groups for whom face-to-face sessions should always be offered where possible? Who/why?
- Are there specific points in the pathway when remote delivery is more suitable e.g. face-to-face to start with and then remote later?
- Do groups typically become friends? How important is the social aspect of the programme in terms of building friendships amongst service users and peer support? [explore social support, isolation, connectedness].
- How is this impacted by remote delivery? What are the barriers to service users building friendships/social support when delivery is remote? Is there anything you have learnt that helps to build this even when they can’t meet face-to-face?
- What about the relationship/rapport between you and the people you support? How is this impacted by remote delivery? [explore social support, isolation, connectedness]. What are the barriers to this? Is there anything you have learn that helps to build this even when you can’t meet face-to-face?
- Do you have a view on how engagement with the programme has been impacted by moving to remote delivery? Better, worse, the same?
- Have you noticed any groups of people who have struggled to engage with the remote delivery? Who? Are these the same groups who are more difficult to engage with generally? What types of problems/difficulties have people experienced engaging with remote delivery? What additional support do these groups need?
- Are there any particular types of exercise session that are more difficult to deliver remotely e.g. because it is hard to demo the exercise, to make sure *service users* are doing it safely, or because that particular group of *service users’* needs the face-to-face support?
- What type of support do people need to be able to able to successfully engage with remote delivery [*explore practical things such as IT, gym equipment but also social/emotional support*]?
- Are there any sessions that you think should always be delivered face-to-face if possible? Why?
- Do you have any thoughts on what service user characteristics or factors influence whether they will stay on the programme until the end (16 weeks)? Has remote influenced this at all (i.e. made it more or less likely)? In what way?
- How have you found remote delivery? How has it made you feel? Do you think this is the right direction for the programme to be going in the future?
- What are your thoughts on centralising remote content (i.e. providing live/ recorded sessions to service users in all areas via a single platform)? What impact might this have on the local ‘character’ of the programme? How important is this?
- What have you learnt about remote delivery that you wish you had known at the start?
- Is there any additional support (management, training, equipment) that you need to help you to deliver aspects of the programme remotely?
- What are your hopes for the future of NERS? What changes would you like to see?

**Debrief**

Participants will be thanked for their participation and asked if they have any questions. They will be asked for feedback on the activity and about how they felt during the focus group.

**[SERVICE USER INTERVIEW SCHEDULE]**


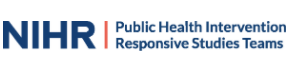


NOTE – This semi-structured schedule covers the topics that will be included in the interviews, but the **exact wording and order will vary depending on the responses**. This script includes a range of possible questions and areas of interest. The actual interview may be shorter or more focused on certain areas, based on the development of the research process and participants’ responses. Additionally, questions should be phrased as open-ended to elicit further information.

Later interviews are likely to include additional areas which arise from analysis of the first set of transcripts. Sensitive use of prompts is likely to encourage participants to expand their opinions on any of the topics in the schedule, looking particularly for anything that might have a story attached to it.

**Introduction**

Confirm with participant that they have read and understood the Participant Information Sheet and give them the opportunity to ask questions. Remind participant that the interview will be recorded (and INITIATE RECORDING).

Re-state the aim of the interview, explain to participant that the goal is to talk and that they are welcome to provide thoughts and views even where these are newly formed and have not been fully thought through or developed. Clarify that all data will be anonymized using a unique participant number rather than their names, and that they are able to withdraw at any point. Ask if participant has any other questions.

**Referral and enrolment**

Explain to participant that the first section will be focused on their experience of the referral and enrolment to NERS.

- Who referred you to NERS? How was this discussion started and by whom? Why do you think you were referred? What were you hoping to get out of the programme? [explore improvements in health, wellbeing, and also making friendships]
- How appropriate did you feel NERS was for you and your particular circumstances at this time (i.e. before you’d started)?
- How would you sum up your overall experience of the referral process?
- What were you told about NERS [*including how it would be delivered*] at i) referral, ii) invite letter, iii) by EP at first consultation)? How well did you understand NERS at each of these timepoints? What attracted you to it? Anything you were hesitant/unsure about [*pick up on delivery mode here*]? Anything that was unclear?
- What could be improved here (amount, tone, clarity of info)?
- What led to your decision to join the programme?
- Did you discuss it with anyone? If so, who and what were their thoughts? How supportive were they of your decision?
- Did the referral come at the right ‘time’ for you? What were the benefits as you saw them?
- How did you feel about the way it was going to be delivered? Was this a good fit for you?

**The NERS programme**

***For those on/offered the virtual programme***

- What were your thoughts ahead of the first consultation? [consider not going? explore]. Did you receive any encouragement/support to attend? [explore what received]. What (additional) encouragement/support would have been helpful? What was your experience of the initial consultation?
- What were your thoughts ahead of the first exercise session? [consider not going? explore] Did you receive any encouragement/support to attend? [explore what received]. What (additional) encouragement/support would have been helpful?
- What types of exercise sessions did you first have and how suitable did you feel these were?
- How many weeks (approx.) of face-to-face sessions did you have before the first lock-down (when offer of remote support made)?
- What was your experience of the sessions up until that point? Was the programme what you expected it to be like? Explore in what way it was/wasn’t.
- Did you enjoy these sessions? What did you like most/least? Anything that you thought could be done to improve these face-to-face sessions?
- Were you attending all/most of them? What were the challenges of attending? Anything that would have made attendance easier/more attractive? Anything that has helped to keep you motivated/attending?
- Please tell me about the relationship between you and your EP in these first few weeks (when still face-to-face). Was this important to your engagement/your progress?
- What about support from others outside of the programme? Did family/partner/friends support and/or encourage you? [explore]
- How much support did you get from your EP during this time? Was the amount/type of support offered right for you? Could you have managed with less? Did you need more?
- Did you make any friends during these first few weeks (when still face-to-face)? Did you feel that making friends and having their support would be an important factor for you in terms of helping you to stay motivated and achieve your goals?
- At the start of the first lockdown, the NERS programme was suspended but then you were contacted to ask you if you would like to continue the programme in remote form. What were you told about what the new programme would be like/how it would be ‘delivered’?
- What was your initial response to this? What did you see as the pros and cons of each?

[cons of virtual/pros face-to-face: unsuitable home environment, IT literacy, loss of social support/connection with EP and other service users]

[pros of virtual/cons of face-to-face: e.g. convenience (incl no travel time), no charge for sessions, no cost of travel or parking; more privacy (esp if self-conscious exercising), more choice as not restricted by what is offered locally, gym/leisure centre environment unfamiliar/intimidating].

- What decision did you make and why?

*For those who postponed ask:*

- What might have encouraged you to continue? Anything that the programme could/should have put in place?
- Have you kept in touch with anyone else who was on the programme? [If so, explore in what way, e.g. whether support each of to be active, emotional support, friendship etc. If not, explore why think this is? E.g. due to programme being cut short or covid restrictions on face-to-face interaction]
- Do you plan on resuming the programme at some point? What needs to be in place for this to happen?
- For the NERS programme, do you think the different types of delivery (face-to-face and remote) are more/less suitable for different types of people? Who are they more suitable for? Who are they less suitable for?

*For those who continued ask:*

- Tell me about your experience of the remote sessions/activities in general? What did you get involved with? Did you enjoy the sessions/activities? What did you like most/least? Were they what you expected?
- Did you have any live online exercise sessions? If so, what was the first session you experienced like? How did you feel beforehand? Were you given enough information/support from the NERS programme/your EP to prepare you for this (probe: brief summary of support)?
- Was access to suitable devices and/or internet an issue for you at all? What about your knowledge/confidence in connecting to the sessions online? What about exercise equipment, or access to appropriate environment (space/privacy)?
- Is there anything that you feel the programme could/should have done to support you here? What would have made things easier?
- Was your relationship with your EP affected by the change to remote delivery? If so, how? [explore social support, isolation, connectedness]
- How much support did you get from your EP (to engage with the programme and attend sessions) during this time? Was the amount/type of support offered right for you? Did you need more/could you have managed with less? Could this have been improved? How?
- What about support/encouragement from family/partner/friends? Was anyone at home with you when you were doing the live online sessions? Did that help? Or cause embarrassment/distraction/discomfort?
- What about others on the programme? Were any friendships that you had affected by the change to remote delivery? In what way? [social support, isolation, connectedness] Has remote delivery made it easier or more difficult to make/maintain friendships?
- [If have made friends]: Are these friendships limited to the exercise sessions or do you have contact with them outside of these? Do you expect these friendships to continue after the end of the programme? If so, is this likely to involve continuing to support each other to be active? In what way?
- Did you manage to attend two sessions per week? [explore what level of engagement there was]. Did you complete the 16-week programme? What were the challenges of attending? Anything that has helped to keep you motivated/attending?
- Do you think you made as much progress as you would have done if the sessions had all been face-to-face? Did you feel they were suitable for you? How could they be improved?
- Having experienced both face-to-face and remote sessions, would you say you have a preference? What are their pros and cons?

[cons of virtual/pros face-to-face: unsuitable home environment, IT literacy, loss of social support/connection with EP and other service users]

[pros of virtual/cons of face-to-face: e.g. convenience (incl no travel time), no charge for sessions, no cost of travel or parking; more privacy (esp if self-conscious exercising), more choice as not restricted by what is offered locally, gym/leisure centre environment unfamiliar/intimidating].

- Have you had your 16-week assessment? If so, was this held remotely? What was your experience like? How did it compare to the initial assessment that you had face-to-face? If not, why did you not attend?
- How prepared do/did you feel to continue being active after the 16 weeks? Have you been able to maintain your activity levels since? Tell me more about this? Why do you think this is? What has helped/hindered this?
- If there were no restrictions in place, might you still choose remote over face-to-face sessions? Do you think the different types of delivery are more/less suitable for different types of people and at different stages in the programme? Are there some parts of the programme that should always be face-to-face (and why)?

***For those on the modified programme***

- What were your thoughts ahead of the first consultation? [consider not going? explore] Did you receive any encouragement/support to attend? [explore what received]. What (additional) encouragement/support would have been helpful? What was your experience of the initial consultation?
- What were your thoughts ahead of the first exercise session? [consider not going? explore] Did you receive any encouragement/support to attend? [explore what received]. What (additional) encouragement/support would have been helpful?
- What types of exercise sessions were you offered? [in terms of delivery mode i.e. face to face only, remote only or a mixture of the two] [establish if know why this format was offered i.e. level of covid alert and/or clinical vulnerability]

- What types of exercise sessions have you had so far? What has been your experience of these and how suitable for you have you found these? [explore content of sessions and delivery mode]
- Is the programme what you expected it to be like? Explore in what way it is/isn’t
- [For those that only experienced only one type of delivery mode, establish whether they had the opportunity to have the other and if so, why they didn’t take this up] [NB even for those offered face-to-face, virtual may be available in some areas on a discretionary basis; establish whether they knew if virtual sessions were available to them]
- [Explore the pros and cons of face-to-face and virtual; if individual has experienced only one type of delivery, explore *perceived* pros/cons of the other and whether this was an influencing factor in them having had just the one]

[cons of virtual/pros face-to-face: unsuitable home environment, IT literacy, loss of social support/connection with EP and other service users]

[pros of virtual/cons of face-to-face: e.g. convenience (incl no travel time), no charge for sessions, no cost of travel or parking; more privacy (esp if self-conscious exercising), more choice as not restricted by what is offered locally, gym/leisure centre environment unfamiliar/intimidating].

- Do you enjoy the sessions? What do you like about them the most/least?
- Have you managed to attend two sessions per week? [explore what level of engagement there was]. Did you complete the 16-week programme? What have been the challenges of attending? Anything that has helped to keep you motivated/attending?
- Did you feel the sessions were suitable for you? How could they be improved?

***If experienced remote delivery (NB some of the Qs in this section may be more suitably incorporated with those above if remote delivery experienced):***

- Tell me about your experience of the remote sessions/activities in general? What have you got involved with? Have you enjoyed the sessions/activities? What have you liked the most/least?
- Have you had any live online exercise sessions? If so, what was the first session you had like? How did you feel beforehand? Were you given enough information/support from the NERS programme/your EP to prepare you for this?
- Has access to suitable devices and/or internet been an issue at all? What about your knowledge/confidence in connecting to the sessions online? What about exercise equipment, or access to appropriate environment (space/privacy)?
- Did you need/get any help in setting up the online sessions? Do you feel that you had the appropriate support to attend these sessions?
- Was anyone at home with you when you were doing the live online sessions? Did that help? Or cause embarrassment/distraction/discomfort?

**All**

- Did you gain anything from being on the programme [acknowledge that only on it for a short time]? If so, what was this? [explore physical health, mental health, friendship etc]
- Did the enjoy the sessions? Any impact on your feelings of wellbeing/mood?
- What about since the programme? Any lasting benefits?

- Please tell me about the relationship between you and your EP. Has this been important to you/your progress?
- Has the way in which the programme has been delivered affected the ability of you and your EP to form a supportive relationship?
- How much support have you had from your EP? Is the amount/type of support offered right for you? Could you have managed with less? Did you need more? Could this be improved? How?
- What about support from others outside of the programme? Did family/partner/friends support and/or encourage you? [explore]
- Have you made any friendships with others on the programme so far? Do you think that making friends with others on the programme is important in terms of helping you to stay motivated and achieve your goals
- Has the way in which the programme has been delivered affected your ability to make friends?
- [If have made friends]: Are these friendships limited to the exercise sessions or do you have contact with them outside of these? Do you expect these friendships to continue after the end of the programme? If so, is this likely to involve continuing to support each other to be active? In what way?

- If there were no restrictions in place, what type of delivery would you choose for yourself: remote only, face-to-face only or a mixture of the two? What is your reasoning here?
- Do you think the different types of delivery are more/less suitable for different types of people and at different stages in the programme? Are there some parts of the programme that should always be face-to-face?
- Do you feel you would have made more or less progress as you have to date if the sessions had been delivered in a different way?
- What changes would you like to see for NERS in the future, particularly in terms of how it is delivered?
- If providing some content digitally is something that is maintained going forward (i.e. live online sessions, recorded sessions), how important do you think it is that these are offered by EPs that the service users you know i.e. continuity? How important is it that they have a ‘local’ feel?

**Conclusion and debrief**

Explain to participant we have finished the questions.

- How have you felt throughout the interview?
- Is there anything else you’d like to add?
- Is there anything you were expecting me to ask you that I didn’t? Were you shocked by any questions?
- How could we improve the interview?

Thanks.

**[DECLINER INTERVIEW SCHEDULE]**


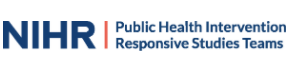


NOTE – This semi-structured schedule covers the topics that will be included in the interviews, but the **exact wording and order will vary depending on the responses**. This script includes a range of possible questions and areas of interest. The actual interview may be shorter or more focused on certain areas, based on the development of the research process and participants’ responses. Additionally, questions should be phrased as open-ended to elicit further information.

Later interviews are likely to include additional areas which arise from analysis of the first set of transcripts. Sensitive use of prompts is likely to encourage participants to expand their opinions on any of the topics in the schedule, looking particularly for anything that might have a story attached to it.

**Introduction**

Confirm with participant that they have read and understood the Participant Information Sheet and give them the opportunity to ask questions. Remind participant that the interview will be recorded (and INITIATE RECORDING).

Re-state the aim of the interview, explain to participant that the goal is to talk and that they are welcome to provide thoughts and views even where these are newly formed and have not been fully thought through or developed. Clarify that all data will be anonymized using a unique participant number rather than their names, and that they are able to withdraw at any point. Ask if participant has any other questions.

**Referral and enrolment**

Explain to participant that the first section will be focused on their experience of the referral and enrolment to NERS.

- Who referred you to NERS? How was this discussion started and by whom? Why do you think you were referred?
- How appropriate did you feel NERS was for you and your particular circumstances?
- What were you told by this person about NERS (including how it would be delivered, what to expect)? How well did you understand NERS after this explanation? What were your initial thoughts? Were you interested? How did you respond? How could this initial explanation have been improved?

*For those who indicated initial interest and were contacted by NERS programme:*

- How were you first contacted by the NERS programme? In what way? What was this communication (invite letter and/or phone call) like? How could this have been improved?

*All:*

- What led to your decision *not* to join the programme? What were the reasons behind this?
- Did you have the appropriate support and resources to engage in the programme if you had wanted/been able to?
- What (if any) were the benefits you could see of joining at the time of the referral?
- What concerns or reservations did you have?
- How did you feel about the way it was going to be delivered? Was this a good fit for you (Probe: aspects that were a good/poor fit and why)
- Did you discuss it with anyone? If so, what were their thoughts? How supportive were they of your decision?
- How did the timing of the referral impact on your decision? (explore responses)
- Are there any circumstances in which you would have accepted the referral?

**Conclusion and debrief**

Explain to participant we have finished the questions.

- How have you felt throughout the interview?
- Is there anything else you’d like to add?
- Is there anything you were expecting me to ask you that I didn’t? Were you shocked by any questions?
- How could we improve this interview?

Thanks.
