# Supplementary material for: Adaptations to the welsh national exercise referral scheme during the COVID-19 pandemic: a qualitative study exploring the experiences of service users and providers and supplementary out-of-pocket cost analysis
Source: BMC Public Health. 2025 Feb 1;25:406. doi: 10.1186/s12889-025-21502-3 (PMC11786398; doi:10.1186/s12889-025-21502-3)
Supplement: Supplementary file 4 — Supplementary Material 4 [file 12889_2025_21502_MOESM4_ESM.docx]

**Table 1. Characteristics of service providers**

| **ID** | **Gender** | **Ethnicity** | **Age** | **Qualifications** | **Total years’ experience as exercise professional** | **LA deprivation*** |
| --- | --- | --- | --- | --- | --- | --- |
| F1 | F | W | 58 | Level 3 & level 4 | 11.5 | Low |
| F2 | F | W | 48 | Level 3 & level 4 | 30 | Low |
| F3 | F | W | 49 | Level 3, level 4 & degree | 10 | Low |
| F4 | F | W | 36 | Level 3, level 4 & degree | 12 | Low |
| F5 | F | W | 43 | Level 4 | 22 | Low |
| F6 | F | W | 57 | Level 4 | 6 | Low |
| F7 | F | W | 47 | Level 3 & level 4 | 14 | High |
| F8 | F | W | 42 | Level 4 | 10 | Low |
| F9 | F | W | 39 | Level 3, level 4 & degree | 14 | Low |
| M1 | M | W | 42 | Level 3 & level 4 | 4 | High |
| M2 | M | W | 31 | Level 4 | 15 | High |
| M3 | M | W | 47 | Level 4 | 14 | High |
| M4 | M | Prefer not to say | 33 | Level 3 | 5 | Low |
| M5 | M | W | 34 | Level 3 & level 4 | 6 | Low |
| M6 | M | W | 55 | Level 3, level 4 & degree | 13 | High |
| M7 | M | W | 32 | Level 3 & level 4 | 3.5 | Low |
| M8 | M | W | 39 | Level 3 & level 4 | 7 | High |
| M9 | M | W | 54 | Level 4 | Missing | Low |
| M10 | M | W | 37 | Level 3 & level 4 | 13 | Low |

*Based on the percentage of Lower Super Output Areas within each local authority which are ranked in the most deprived 50% of LSOAs in Wales (low is below the national average (50%) and high is above). M= male, F= female, LA= local authority. Level 3 and Level 4 are advanced qualifications recognised by the fitness industry; Level 4 qualifications are condition specific e.g. cancer rehab, falls prevention.

**Table 2. Characteristics of service users and the individual who declined WNERS**

| **ID** | **Participant type** | **Status** | **Pathway** | **Gender** | **Ethnicity** | **Age** | **LA** | **LA deprivation*** |
| --- | --- | --- | --- | --- | --- | --- | --- | --- |
| F10 | SU | Remote programme (virtual; completed) | Generic | Female | White | 61 | Anglesey | Low |
| F11 | SU | Declined remote programme | Generic | Female | White | 59 | Pembrokeshire | Low |
| F12 | SU | Remote programme (home programme; completed) | Pulmonary | Female | White | 83 | Gwynedd | Low |
| F13 | SU | Remote programme (virtual; completed) | Generic | Female | White | 73 | Anglesey | Low |
| F14 | SU | Remote programme (virtual; completed) | Falls prevention | Female | White | 60 | Swansea | Low |
| F15 | SU | Remote programme (virtual; completed) | Back care | Female | White | 51 | Pembrokeshire | Low |
| F16 | SU | Remote programme (virtual; completed) | Cardiac | Female | White | 66 | Bridgend | High |
| F17 | SU | Declined remote programme | Cardiac | Female | White | 76 | Pembrokeshire | Low |
| F18 | SU | Remote programme (virtual; withdrew) | Cancer | Female | White | 74 | Cardiff | Low |
| F19 | D | Declined WNERS referral | Unknown | Female | White | 44 | Unknown | Unknown |
| F20 | SU | Modified (all face-to-face) | Generic | Female | White | 74 | Neath Port Talbot | High |
| F21 | SU | Modified (all face-to-face) | Generic | Female | White | 69 | Wrexham | Low |
| F22 | SU | Modified (all face-to-face) | Cardiac | Female | White | 69 | Powys | Low |
| M11 | SU | Declined remote programme | Cardiac | Male | White | 53 | Swansea | Low |
| M12 | SU | Remote programme (walking challenges; completed) | Generic | Male | White | 51 | Powys | Low |
| M13 | SU | Remote programme (virtual; completed) | Generic | Male | White | 90 | Flintshire | Low |
| M14 | SU | Remote programme (virtual; completed) | Cardiac | Male | White | 70 | Swansea | Low |
| M15 | SU | Declined remote programme | Cardiac | Male | White | 66 | Wrexham | Low |
| M16 | SU | Standard programme (withdrew) | Generic | Male | White | 80 | Ceredigion | Low |
| M17 | SU | Remote programme (virtual; withdrew) | Generic | Male | White | 76 | Swansea | Low |
| M18 | SU | Modified (all virtual) | Generic | Male | White | 62 | Monmouthshire | Low |
| M19 | SU | Modified (all face-to-face) | Generic | Male | White | 85 | Denbighshire | Low |

*Based on the percentage of Lower Super Output Areas within each local authority which are ranked in the most deprived 50% of LSOAs in Wales (low is below the national average (50%) and high is above). SU = service user, D = decliner, M= male, F= female, LA= local authority

**Table 3. Service user time with the NERS and mean number of sessions attended per week by delivery mode**

| **Domain** | **Statistic** | **Face-to-face only** | **Virtual only** | **Both** | **Overall** |
| --- | --- | --- | --- | --- | --- |
| Sample size | N | 11 | 1 | 9 | 21 |
| Time with the NERS | Mean (weeks) | 63 | 17 | 62 | 61 |
|  | SD (weeks) | 116 | - | 105 | 106 |
|  | Time in the Welsh NERS: 0-16 weeks (people) | 7 | 0 | 4 | 11 |
|  | Time in the Welsh NERS: >16 weeks - 1 year (people) | 2 | 1 | 2 | 5 |
|  | Time in the Welsh NERS: ≥1 year (people) | 2 | 0 | 3 | 5 |
| Sessions per week | Mean weekly sessions (sessions) | 1.82 | 2.00 | 1.00 | 1.77 |
| Face-to-face period | Mean weeks attended (weeks) | 63 | - | 42 | 51 |
|  | Mean weekly sessions (sessions) | 1.82 | - | 1.33 | 1.60 |
| Virtual period | Mean weeks attended (weeks) | - | 17 | 20 | 10 |
|  | Mean weekly sessions (sessions) | - | 2.00 | 1.38 | 1.44 |
